# Automated time activity classification based on global positioning system (GPS) tracking data

**DOI:** 10.1186/1476-069X-10-101

**Published:** 2011-11-14

**Authors:** Jun Wu, Chengsheng Jiang, Douglas Houston, Dean Baker, Ralph Delfino

**Affiliations:** 1 Program in Public Health, University of California, Irvine, USA; 2 Department of Epidemiology, School of Medicine, University of California, Irvine, USA; 3 Department of Planning, Policy and Design, School of Social Ecology, University of California, Irvine, USA; 4 Center for Occupational & Environmental Health, University of California, Irvine, USA

## Abstract

**Background:**

Air pollution epidemiological studies are increasingly using global positioning system (GPS) to collect time-location data because they offer continuous tracking, high temporal resolution, and minimum reporting burden for participants. However, substantial uncertainties in the processing and classifying of raw GPS data create challenges for reliably characterizing time activity patterns. We developed and evaluated models to classify people's major time activity patterns from continuous GPS tracking data.

**Methods:**

We developed and evaluated two automated models to classify major time activity patterns (i.e., indoor, outdoor static, outdoor walking, and in-vehicle travel) based on GPS time activity data collected under free living conditions for 47 participants (N = 131 person-days) from the Harbor Communities Time Location Study (HCTLS) in 2008 and supplemental GPS data collected from three UC-Irvine research staff (N = 21 person-days) in 2010. Time activity patterns used for model development were manually classified by research staff using information from participant GPS recordings, activity logs, and follow-up interviews. We evaluated two models: (a) a rule-based model that developed user-defined rules based on time, speed, and spatial location, and (b) a random forest decision tree model.

**Results:**

Indoor, outdoor static, outdoor walking and in-vehicle travel activities accounted for 82.7%, 6.1%, 3.2% and 7.2% of manually-classified time activities in the HCTLS dataset, respectively. The rule-based model classified indoor and in-vehicle travel periods reasonably well (Indoor: sensitivity > 91%, specificity > 80%, and precision > 96%; in-vehicle travel: sensitivity > 71%, specificity > 99%, and precision > 88%), but the performance was moderate for outdoor static and outdoor walking predictions. No striking differences in performance were observed between the rule-based and the random forest models. The random forest model was fast and easy to execute, but was likely less robust than the rule-based model under the condition of biased or poor quality training data.

**Conclusions:**

Our models can successfully identify indoor and in-vehicle travel points from the raw GPS data, but challenges remain in developing models to distinguish outdoor static points and walking. Accurate training data are essential in developing reliable models in classifying time-activity patterns.

## Background

Environmental air pollution has been associated with a variety of adverse health outcomes, including respiratory illness, cardiovascular diseases, pregnancy outcomes, and morbidity [1-5]. The knowledge of where individuals spend time is essential for human exposure assessment of air pollution because air pollutant concentrations may vary significantly by location. Studies have shown that traffic-generated air pollutants such as ultrafine particles can be up to ten times higher inside a vehicle compared to ambient outdoor concentrations because of proximity to vehicle exhaust [6-8]. Outdoor walking and cycling spaces often have lower concentrations of traffic-related pollutants than in-vehicle spaces [9], but often correspond with increased inhalation rates and longer travel durations which could result in a higher dose of air pollutant inhalation [10]. In addition, air pollutant concentrations can be much higher indoors than outdoors for pollutants with predominate indoor sources (e.g. environmental tobacco smoke) and vice versa for pollutants with predominate outdoor sources (e.g. ozone) [11,12]. Accurate characterization of people's time-location patterns significantly reduce errors in exposure estimates in environmental epidemiological studies in which personal exposure is not measured directly and has to be estimated.

Time activity data have traditionally been collected by recall telephone interviews or activity logs recorded by study participants [13,14]. However, these methods are limited by accuracy of recall, reliability, and compliance [15]. Recently, new techniques have been used to collect time-location data, such as the use of portable global positioning system (GPS) devices to track people's time-location or commuting patterns with or without corresponding participant diary information [15-22]. GPS-based tracking presents an enormous opportunity for improving our understanding of the space-time activities of individuals and how they influence environmental exposure and health outcomes. It offers many advantages over traditional methods including near-continuous location tracking, high temporal resolution, and minimum reporting burden for participants [23]. However, barriers exist for extracting accurate time activity patterns for human subjects from raw GPS data because they are not consistently reliable due to errors caused by satellite or receiver issues, atmospheric and ionospheric disturbances, multipath signal reflection, or signal loss or blocking [24]. The multipath problem occurs mainly in urban areas where tall buildings and structures reflect satellite signals many times before they reach a GPS device, leading to GPS coordinate errors [25].

Since GPS datasets are usually very large (e.g., over 5,000 location coordinates per day at a 15-second interval) and are associated with such uncertainties, validated techniques are needed to automatically identify time activities in major microenvironments, such as commuting, indoor, and outdoor locations. The literature on GPS data classification techniques largely comes from the travel behavior and physical activity research fields. A number of studies have developed methods to classify travel activity using only GPS data or the combination of GPS and accelerometer data [26-34]. However, most of these studies collected data and developed models solely for travel activities (e.g. mode of travel, route choice, and distance traveled); some measurements were even conducted at predefined routes or activity schedules. Only a few studies have used GPS to track people's time-activity patterns in free living conditions. Cho et al. [35] collected GPS and self-reported one-week location diary data for 5 research staff (35 person-days of data) for model development and calibration and 34 volunteers (136 person-days of data) for model validation. They successfully developed and validated an algorithm to identify outdoor walking trips that lasted more than 5 minutes in free-living conditions. The authors reported difficulty in identifying walking trips with only GPS information in free living conditions because people often walk very slowly or briskly and make short stops. Unfortunately, the application of the techniques used in these studies in air pollution epidemiological research is limited because people's exposure to air pollution is significantly influenced by activity patterns across multiple indoor, outdoor, and transportation microenvironments. Few studies have evaluated techniques to classify people's continuous time-activity patterns across both travel and non-travel periods based on GPS location data for subjects in free living conditions. To our knowledge, only one study by Adams et al. [36] developed an apportion algorithm for time-location patterns across indoor, outdoor and travel microenvironments based on data from a monitoring system that measured personal air pollution exposure, temperature, and real-time GPS locations. However, this study only tested the algorithm for a single person for four days with known home and workplace locations. In addition, their use of temperature to distinguish indoor from outdoor locations in the winter of Colorado may not be applicable to other regions or seasons when indoor and outdoor temperature does not vary as much.

The purpose of this study was to address a major gap in the literature on air pollution epidemiology - the lack of reliable automated classification techniques to post-process multi-day GPS location tracking data for free living human subjects. We developed and evaluated two automated models that classify GPS tracking data into four major time-activity categories (i.e. indoor, outdoor static, outdoor walking, and in-vehicle travel) that are important in determining people's exposure to traffic-related and other air pollutants [6-12].

## Method

### GPS and Roadway Data

We developed our classification models based on 131 person-days of GPS time activity data collected for 47 participants in the Harbor Communities Time Location Study (HCTLS). Participants were 21-65 years old and were tracked for 3 days from February 19 to June 13, 2008 in the Wilmington area of the City of Los Angeles and the western portion of the City of Long Beach, California [37]. Data were available for 3 days for 37 participants and 2 days for 10 participants. Participants were asked to carry a portable GlobalSat DG-100 GPS device (approximately 227 g) with them during waking hours on the observation days. Concurrently, the participants recorded in an activity log each time they changed location by recording the time, checking whether they were indoors (home, work, school, other), outdoors (walking, biking, other), or in-vehicle (auto, van, or truck, transit, or other), and noting location details. After the logs and the GPS data were retrieved, participant GPS data were reviewed over highly resolved and geographically rectified Digital Ortho Quarter Quads (DOQQ) aerial photography data from the United States Geological Survey using geographical information system (GIS) techniques in ArcGIS software (ESRI, Redlands, CA). Based on the participant logs and DOQQ imagery, prompts were generated for follow-up interviews regarding discrepancies, unclear patterns, and suspected unreported activities. Follow-up interviews were administered 2-5 weeks after the monitored days because of the time required for post-processing GPS data and logistics. Based on feedback from these interviews, the GPS data were finalized and coded into major location categories (e.g., indoor/outdoor at home, school, restaurants, etc.) and travel mode (e.g., biking, walking, automobile, bus, train). Unfortunately these manually-classified data at times do not precisely differentiate indoor vs. near-building outdoor points due to the positional error of GPS data [24]. In instances when it was not possible to consistently distinguish near-building outdoor locations (e.g., patio and sidewalk) from indoor locations, participants were assumed to be indoors unless GPS locations were consistently separated from a building for at least 2-5 minutes, in which case they were classified as outdoor.

Most (92.1%) of the HCTLS data were collected at a 15-second interval, while 4.0%, 0.1% and 3.8% of the data were collected at 1-second, 2 to 14 seconds, and > 15-second intervals, respectively. Since the recording intervals may occasionally shift even with a fixed interval setting in the GPS device, we decided to exclude only those points with no time stamp and data from one participant who had the majority of the data in 1-second interval. Data with speed higher than 200 kilometers per hour (km/h) (0.002% of all the data) were corrected to zero speed because they were determined through mapping and follow-up interviews to be erroneous GPS locations which appeared far from the participant location and were caused by GPS device error or blocked satellite signals.

In addition to the HCTLS data, we collected 21 person-days of supplemental GPS time activity data for three UC-Irvine staff volunteers during December 6-13, 2010. The volunteers were asked to carry with them a GPS device (BT-Q1000XT from QSTARZ; approximately 65 g; recording at a 15-second interval) and to record on a paper diary the following information: the start and end time for their major time activity patterns, mode of travel (outdoor walking or biking, in-vehicle travel by passenger car, bus, or train), and type of location including indoor or outdoor (home, work, school or education, other residence, park and recreational area, food-consumption related location, retailer store, short stops for gas-filling etc.). The BT-Q1000XT was used in this task mainly because it had longer battery life (48 hours) than the DG-100 model used in the HCTLS (17 hours) [24]. Consistent with the manually-classified HCTLS data, these supplemental GPS data were manually classified based on the diary data, GIS verification, and verification by interview. The three staff volunteers were thoroughly instructed on how and when to record the diary logs and all of them were actively involved in the time activity research and knowledgeable of the study aims and possible mistakes that may be encountered by human participants.

There were two major differences between the HCTLS data and the supplemental UCI data. First, we obtained better diary logs in the supplemental dataset than the HCTLS study (particularly for the indoor vs. outdoor differentiation) because of more thorough and careful documentation of short-term events. The HCTLS only classified outdoor points as those that were consistently outdoors for at least 2-5 minutes based on map overlays in GIS software while the supplemental dataset recorded outdoor points at a much higher accuracy of less than 1 minute. In addition, slight differences were observed between the DG-100 used in the HCTLS and the BT-Q1000x used in supplemental data collection; BT-Q1000x had a shorter acquisition time at cold start in an indoor environment, lower rate of data loss, but slightly poorer performance in spatial accuracy [24].

All GPS data were converted to the Universal Transverse Mercator projection (North American Datum NAD 83 and Zone 11 N). In addition to the above GPS data, we obtained roadway data for the study region from the ESRI StreetMap™ North America 9.3 http://www.esri.com. This dataset was bundled with ArcGIS software products and included 2003 TeleAtlas^® ^street data rather than the less-accurate TIGER 2000-based street data [38].

### Model Development and Time-Activity Classifications

We developed a rule-based model and a decision-tree based model to classify time-location patterns. The two types of models were chosen because the rule-based method best utilizes the user's understanding of spatial patterns in the data (time, speed, and spatial relationships of the GPS points) while the decision-tree method requires minimal user-input and has been shown to outperform the other approaches [32].

We focused on four time-activity patterns (i.e. indoor, outdoor static, outdoor walking, and in-vehicle travel) because of their importance in determining air pollution exposures and the availability of data. The in-vehicle travel here refers to travels in passenger vehicles only. Although other time-activity patterns such as biking and travel by bus and subways may also be associated with high levels of exposure to traffic-related air pollutants [39-41], our data contained very limited data on these activities and were not examined in our models. No HCTLS participants traveled in an underground train; only 10 and 3 subjects reported bus travel and biking for a total of approximately 8 and 5 hours, respectively. No subway, bus, or biking activity was reported in the supplemental UCI data.

We did not distinguish indoor static vs. indoor moving conditions because the positional accuracy of GPS data was limited during indoor periods due to obstructed satellite signals from building structures. Our previous research indicates that the positional errors of the GPS devices can be large in typical indoor locations and can be in the range of 0-300 meters for DG-100 depending on the building materials (e.g., wood frame vs. concrete/steel) and surrounding structures [24]. Significant GPS signal loss was also observed indoors in concrete structures or structures surrounded by high buildings [24]. Therefore, we decided not to track participants' moving patterns in an indoor environment. Instead, we asked the study participants (≥21 years old) to place the GPS device on a static location (e.g., counter or desk) nearby while they were indoors at home, school, or work locations; thus most of our indoor data were static points.

### Rule-Based Classification

**Figure 1:**
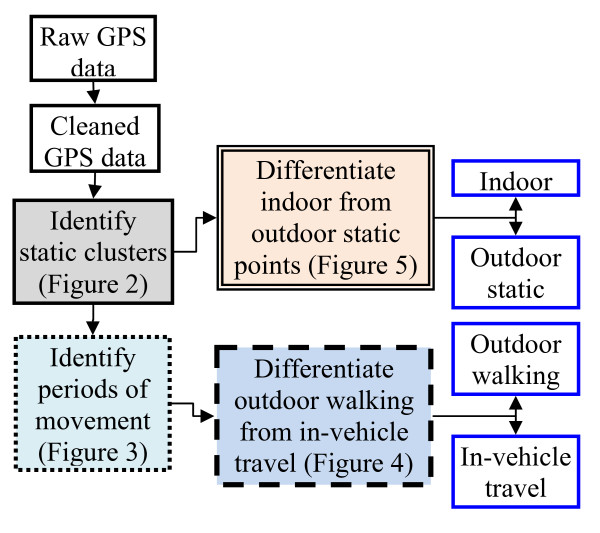
**Overall flow chart**.

We developed the rule-based model using two open source software programs: R (version 2.10.1; R Foundation for Statistical Computing, Vienna, Austria) and PostgreSQL (version 8.4; PostgreSQL Global Development Group). The rule-based algorithm used three major features of the GPS data (i.e., time, speed, and spatial location) to classify GPS data to four major time activity categories: indoor, outdoor static, outdoor walking, and in-vehicle travel (Figure 1). The rules were developed based on logic and the summary statistics of the HCTLS data. For example, the model assumes that in-vehicle travel occurs on roadways and at higher average speeds than outdoor walking and outdoor static conditions. Most of the threshold values were obtained from the summary statistics of each time-activity category in the HCTLS data.

**Figure 2:**
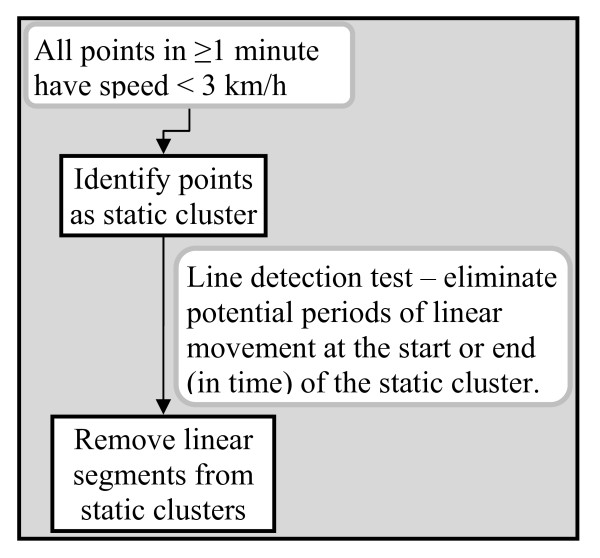
**Identify static clusters**.

First, we identified static clusters and moving periods based on continuity criteria in space and time. If all the points in a minimum of one minute had speed lower than 3 km/h, these points were treated as a static cluster (Figure 2). We further implemented a line detection process to identify the presence of linear alignment of sequential points which could indicate periods of movement within a static cluster. Starting from the first and last points of a cluster in time sequence, we selected three sequential points (moving forward in time from the first point and backward in time from the last point) to test if three consecutive points formed a linear segment. The distance difference was calculated by subtracting (a) the distance from the first sequential point and the third sequential point from (b) the sum of the distance between the first sequential point and the second sequential point and the distance between the second sequential point and the third sequential point. If the distance difference was no more than 1 m, we assumed that the three points formed a line and excluded them from the static cluster. This line detection process proceeded through the sequence of points in each cluster until it detected three continuous points that did not form a line.

**Figure 3:**
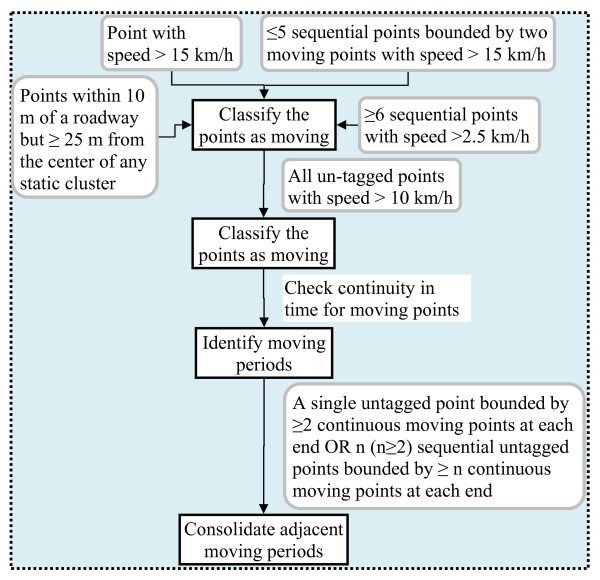
**Identify periods of movement**.

The second step in our rule-based classification was to identify sequential points which represent periods of movement using the following criteria (Figure 3): 1) points with speed above 15 km/h; 2) a maximum of five sequential points bounded by two moving points identified by step 1; 3) a minimum of six continuous points with speed above 2.5 km/h; 4) points within 10 m of a roadway but more than 25 m of the center of any static clusters; and 5) all other untagged points with speed more than 10 km/h. Adjacent moving periods were consolidated into one if there was a single untagged point with at least two continuous moving points before and after this point or there were n (n≥2) continuous untagged points with a minimum of n continuous moving points before and after them.

**Figure 4:**
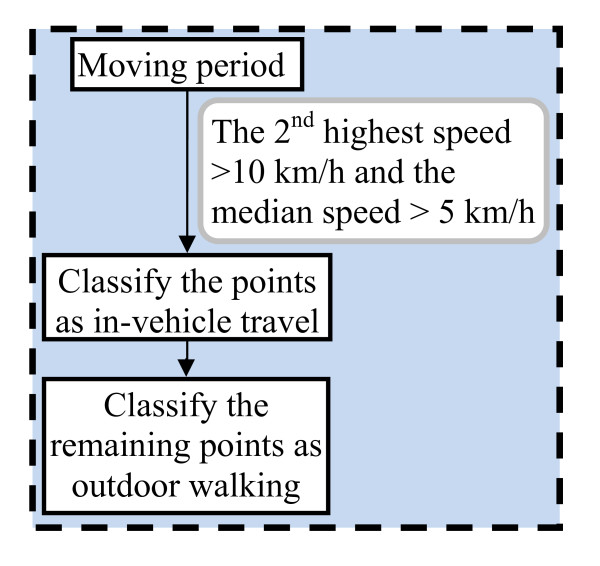
**Differentiate outdoor walking from in-vehicle travel**.

**Figure 5:**
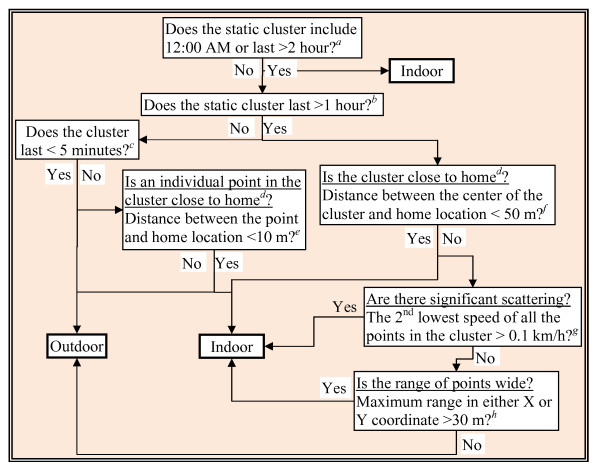
**Differentiate indoor from outdoor static points**. ^a^We found that 99.5% of outdoor static clusters lasted less than 2 hours. ^b^About 2% (number) of outdoor static clusters (accounted for 21% of the total outdoor static time) lasted 1-2 hours. ^c^We found 77% of all the outdoor static clusters that satisfied the upper level rules (the rules above this criterion) lasted less than 5 minutes. ^d^A home location was detected if the cluster included 12:00 AM or lasted for more than 24 hours assuming that the participants were at their home under such conditions. ^e^The spatial accuracy was 10 m for the GPS device without Wide Area Augmentation System. Thus the GPS points within 10 m of home are more likely indoor points. ^f ^We used 50 m as an approximate size of an apartment. ^g^The 2nd lowest speed of all the outdoor static cluster was 0 and 5% (number) of the indoor static cluster (accounted for approximately 15% of the total indoor time) was more than 0.1 km/h. ^h^About 64% of the indoor clusters satisfied this rule with only 2 of 13 outdoor clusters being misclassified.

We further refined the moving classification by differentiating walking from in-vehicle travel periods using speed as a key parameter (Figure 4). A moving period was identified as in-vehicle travel if the second highest speed of the moving period was above 10 km/h and the median speed was above 5 km/h. We used a number of criteria to distinguish outdoor from indoor periods, including duration and time span of a static cluster, distance between a static cluster and a participant's home location, speed, the geographic range of the points in the cluster, and distance among critical points in the cluster (i.e. start, end, and middle point in time sequence) (Figure 5). The criteria were developed by analyzing the statistics of the manually-classified GPS data. For instance, we observed that indoor points occurred more often in static clusters that lasted longer (Additional file 1, Table S1), were closer to home, scattered somewhat more diversely (indicated as higher speed or wider geographic range of points), and showed no apparent line pattern between sequential points. A home static cluster was roughly detected by checking whether the cluster included 12:00 AM or lasted for more than 24 hours, assuming that the participants were at their home under such conditions. We did not attempt to classify other types of locations (e.g. work, school, shopping) because without additional data we have to make many assumptions on the patterns of these activities, which may introduce a lot of uncertainties.

### Random Forest Classification

As an alternative to the rule-based algorithm, we applied a machine learning model, random forest [42], to classify the GPS data into different time activity categories. Here, a forest refers to a constellation of many decision tree models. Because a forest consists of many trees, it is more stable and less prone to prediction errors as a result of data perturbations [42]. Random forest is considered one of the most accurate general-purpose learning techniques available [43] and has been already widely used in bioinformatics [44,45]. Random forest creates multiple classification and regression (CART) trees, each trained on a bootstrap sample of the original training data and searches across a randomly selected subset of input variables to determine the split. Each tree in the forest gives or "votes" for a classification (e.g. time activity category). The forest chooses the classification having the most votes over all the trees in the forest.

In this study, we used an R interface in Waikato Environment for Knowledge Analysis (WEKA) 3.6 software, a popular machine learning workbench developed by researchers in University of Waikatoi [46,47]. We examined the following types of variables: acceleration rate, speed, distance difference, and distance ratio. Acceleration was calculated as the change in speed between a given point and the previous sequential point. The distance difference was calculated for any three sequential points as we described above for the rule-based model. The distance ratio was calculated for a series of sequential points as the ratio between (a) distance between the first sequential point and the last sequential point in the series and (b) the sum of the distance for all line segments formed by sequential points in the series. Speed, distance difference, and distance ratio variables were calculated under various averaging time intervals ranging from 2 to 60 minutes and centered at each GPS point. Speed and distance difference were expressed as minimum, median, and maximum values. Standard deviation was also calculated for distance difference. Final variables were selected by checking the variable importance index (a measure of the relative importance of the variables) generated from the model diagnostics and the correlations among the variables. We first selected 20 most important variables that were not highly correlated with each other (r < 0.75). Then we run the model again with these variables and selected the most important variables with the p-value of the z-score < 0.001 for all the variables. Sensitivity tests were conducted for the maximum depth of a tree and the number of trees. Two separate models were developed based on the HCTLS data (hereafter HCTLS random forest model) and the supplemental UCI data (hereafter UCI random forest model). For both models, we randomly selected a subset of indoor points (N = 30,000 for the HCTLS data and N = 5,000 for the UCI data) in addition to all outdoor static, outdoor walking, and in-vehicle travel points in the original datasets as training data.

### Model Evaluation

Time activity classifications of GPS data based on model predictions were compared to those from manually-classified data for both the HCTLS and the supplemental datasets. There is no "gold-standard" against which to compare the classification of GPS-derived time activity data. In this study we used the manually-classified time activity classifications as the basis of model evaluation because these time activity codes were developed based on extensive quality assurance checks that involved careful inspection of real-time GPS locations in GIS using map overlays, comparison with participant activity logs, and clarifications from follow-up interviews with participants and staff. We examined the sensitivity (the ability of the model to identify specific cases), specificity (the ability of the model to identify non-cases), and precision (the proportion of predicted cases that are correctly real cases) of model prediction for each time activity category. Both the rule-based and the random forest models were evaluated against the HCTLS data and the supplemental UCI data. The two random forest models were evaluated using a repeated 10-fold cross validation method that has been recommended for model selection [48,49]. More specifically, the dataset was randomly split into 10 mutually exclusive subsets of equal size. The random forest model was trained on 9 subsets and validated on the remaining 1 subset of data; the procedure was repeated for 10 times. The reported validation results were the averages from the 10-folds testing. We further evaluated the capability of the random forest models in predicting new data by applying the HCTLS model to the UCI data and the UCI model to the HCTLS data.

## Results

We obtained 406,261 GPS data points from the HCTLS data after exclusion of points with no time stamp (N = 609) and data from one participant who had most of GPS recordings (N = 16,887) at a one-second interval. Indoor, outdoor static, outdoor walking and in-vehicle travel accounted for 83.4%, 6.1%, 3.3% and 7.2% of the data, respectively, based on manually-classified time activity classifications. Based on the HCTLS data, the median duration of periods in each time activity category was 41, 2, 5, and 9 minutes for indoor, outdoor static, outdoor walking, and in-vehicle travel, respectively (Additional file 1, Table S1). The maximum duration of the indoor clusters was 2.2 days (confirmed through our follow-up interview).

Table 1 shows classification results from the rule-based model. The rule-based model classified indoor and in-vehicle travel points reasonably well for the HCTLS data (Indoor: sensitivity = 84%, specificity = 82%, and precision = 96%; in-vehicle travel: sensitivity = 72%, specificity = 99%, and precision = 89%). We observed moderate performance of the model on outdoor static and outdoor walking predictions for the HCTLS data although the precision was very low for the outdoor static predictions (17.6%). For the supplemental UCI data, the model performed better for indoor, outdoor static, and in-vehicle travel predictions, but worse for outdoor walking predictions. For both datasets, outdoor static was the largest category of misclassifications for indoor, outdoor walking, and in-vehicle travel points, while most of the misclassification of outdoor static points was in the indoor category.

**Table 1:** Time activity classification using the rule-based model*^a^*

		Indoor (modeled)	Outdoor static (modeled)	Outdoor walking (modeled)	In-vehicle travel (modeled)	Sensitivity*^b^*	Specificity*^c^*	Precision*^d^*
Model evaluation against the HCTLS data	Indoor (coded)	284830	9840	1002	1362	84.1%	81.9%	95.9%
	
	Outdoor static (coded)	51991	12901	2336	5994	51.7%	84.2%	17.6%
	
	Outdoor walking (coded)	1102	1145	9096	827	68.4%	99.2%	74.7%
	
	In-vehicle travel (coded)	778	1073	857	21127	72.1%	99.3%	88.6%

Model evaluation against the supplemental UCI data	Indoor (coded)	103930	5430	134	144	94.8%	82.6%	98.2%
	
	Outdoor static (coded)	1558	2298	169	214	54.2%	94.4%	26.0%
	
	Outdoor walking (coded)	284	764	646	193	34.2%	99.6%	57.4%
	
	In-vehicle travel (coded)	114	336	176	4512	87.8%	99.5%	89.1%

*^a^*The rule-based model was developed based on logic and the summary statistics of manually-classified time activity classifications of HCTLS data.

*^b^*Sensitivity was calculated as true positive estimation/(true positive estimation + false negative estimation).

*^c^*Specificity was calculated as true negative estimation/(true negative estimation + false positive estimation).

*^d^*Precision was calculated as true positive estimation/(true positive estimation + false positive estimation).

Table 2 shows classification results from the random forest models with ten trees with a maximum depth of three for each tree. Based on variable importance index and correlation analysis, we selected the following five variables in the final models (by ranking of the variable importance index): maximum speed in 4 minutes, maximum speed in 60 minutes, median speed in 30 minutes, maximum distance difference in 6 minutes, and maximum distance difference in 30 minutes. Since the number of predictor variables was small and little difference was found in the results of 10, 15, and 20 trees (unpublished data), we decided to report 10-tree model results in this paper. Sensitivity tests showed that the maximum depth of trees significantly influenced the model performance, particularly for the 10-fold cross validation results (Additional file 1, Table S2). No restrictions on the depth of the trees generated superb cross validation results with precision, specificity, and precision measures all above 88% in the two random forest models (Additional file 1, Table S2). However, when the models were applied to predict new data (e.g. HCTLS model to predict UCI data and vice versa), the performance of the models degraded remarkably (Additional file 1, Table S2), likely due to model over-fit when the trees grow large. Therefore, we reported the results using a maximum depth of three for each tree.

**Table 2:** Time activity classification using the random forest models*^a^*

			Indoor (modeled)	Outdoor static (modeled)	Outdoor walking (modeled)	In-vehicle travel (modeled)	Sensitivity*^b^*	Specificity*^c^*	Precision*^d^*
HCTLS random forest model*^e^*	10-fold cross validation*^f^*	Indoor (coded)	21959	6177	1604	260	73.2%	81.8%	64.1%
		
		Outdoor static (coded)	9777	9749	3596	1830	39.1%	81.7%	42.3%
		
		Outdoor walking (coded)	1052	1820	9931	477	74.8%	92.9%	62.5%
		
		In-vehicle travel (coded)	1475	5307	750	21762	74.3%	96.2%	89.4%
	
	Evaluation against the full UCI dataset	Indoor (coded)	82874	26278	170	314	75.6%	91.6%	98.9%
		
		Outdoor static (coded)	850	3137	35	214	74.1%	76.2%	10.1%
		
		Outdoor walking (coded)	101	993	555	244	29.3%	99.8%	67.7%
		
		In-vehicle travel (coded)	0	515	60	4562	88.8%	99.3%	85.5%

UCI random forest model*^g^*	10-fold cross validation*^f^*	Indoor (coded)	3978	860	120	42	79.6%	99.0%	97.3%
		
		Outdoor static (coded)	109	3285	471	371	77.5%	90.2%	73.6%
		
		Outdoor walking (coded)	0	170	1313	410	69.4%	94.4%	62.1%
		
		In-vehicle travel (coded)	0	146	210	4781	93.1%	92.6%	85.3%
	
	Evaluation against the full the full HCTLS dataset	Indoor (coded)	153216	54796	128829	1894	45.2%	92.8%	96.9%
		
		Outdoor static (coded)	3519	6590	13015	1828	26.4%	84.5%	10.0%
		
		Outdoor walking (coded)	320	725	11840	395	89.2%	63.0%	7.5%
		
		In-vehicle travel (coded)	999	3464	3550	21281	72.6%	98.9%	83.8%

*^a^*The results reported here came from random forest models with 10 trees and a maximum depth of 3 for each tree.

*^b^*Sensitivity was calculated as true positive estimation/(true positive estimation + false negative estimation).

*^c^*Specificity was calculated as true negative estimation/(true negative estimation + false positive estimation).

*^d^*Precision was calculated as true positive estimation/(true positive estimation + false positive estimation).

*^e^*The model was developed based on all outdoor static, outdoor walking, in-vehicle travel and randomly selected 30,000 indoor points

*^f^*The reported validation results were the averages from repeated 10-fold cross validation.

*^g^*The model was developed based on all outdoor static, outdoor walking, in-vehicle travel and randomly selected 5,000 indoor points from the supplemental UCI data.

From the 10-fold cross validation results, we found similar performance of the random forest models compared to the rule-based model except somewhat degraded performance of the HCTLS model for indoor predictions and better performance of the UCI model for outdoor static and outdoor walking predictions. For new data prediction, the HCTLS model performed similarly as the rule-based model in predicting UCI data. However, the UCI model performed worse than the rule-based model in predicting HCTLS data, likely because of the very small and non-representative training dataset for the development of the UCI model. Similar to the rule-based model, we found that the HCTLS model most frequently misclassified indoor, outdoor walking, and in-vehicle travel to outdoor static while outdoor static to indoor category. The misclassification pattern was different for the UCI model, again likely due to the small and non-representative training data.

## Discussion

We developed, evaluated, and compared two models to classify time-location patterns based solely on the publicly-available roadway data and raw GPS data (three-dimensional location data and the corresponding time stamps) from participants under free living conditions. To our knowledge, this is one of the first studies that developed models to systematically classify human time activity patterns for travel and non-travel activities based on raw GPS tracking data for use in air pollution epidemiological studies. Three major strengths of the study include the use of extensive manually-classified time-activity data from human participants under free living conditions for model development, the comparison of two models in classifying time activity patterns, and additional model validation using supplemental data which were carefully collected and coded. The reasonably good performance of the models indicates feasibility of using these models to reliably batch-process GPS tracking data from free living human subjects.

Air pollution epidemiological studies are increasingly using GPS to track time activity patterns of human subjects. However, few studies have used the GPS-derived time activity information extensively in exposure assessment and epidemiological analysis; rather, most prior studies rely on questionnaire-reported time activity patterns in the analysis. Lack of reliable methods to mine raw GPS data may be one of the major reasons for the limited and crude classification of these data. For instance, a Canadian study assigned all GPS points within 350 m of a residence as home and 400 m of a work place as work, and did not differentiate indoor from outdoor environments [50]; such an approach could lead to substantial exposure misclassification. A growing body of transportation and physical activity literature has begun to provide insights into methods for automating the classification of GPS to identify different travel modes during periods of travel [26-34]. However, since our focus is to develop and evaluate procedures to classify GPS data for travel and non-travel periods given the paucity of applicable methods in the air pollution epidemiological literature, we do not provide a comprehensive overview of the transportation and physical activity literature.

Overall we found no striking differences in the performance of the rule-based model and the random forest models. Both models have advantages and disadvantages. The rule-based model features high flexibility and easy-to-interpret results, but the effort involved in the model development is substantial and may require a lot of additional data and resources. The random forest model is easy to use and requires minimum user interference, but it faces a potential over-fitting problem if not properly trained and difficulty in results interpretation. In terms of computational time, random forest was faster than the rule-based model (e.g. it took approximately one hour for the rule-based model to predict the HCTLS data while < 30 seconds for the random forest to build the model for the same dataset on a 64-bit computer with 16 GB of memory and a 2.93 GB Intel^® ^Core™ i7 Quad Core Processor). However, since random forest model is purely data driven, it may be more severely affected by biased or unrepresentative training data than the rule-based model. In fact, we observed poor model performance when the UCI random forest model was applied to predict the HCTLS data. With high quality training data, random forest model should be a good choice to quickly identify important predictor variables, develop exploratory models, or even final model based on its performance.

For the rule-based model, we observed similar model performance for indoor and outdoor static predictions between the HCTLS data and the supplemental UCI data. We observed improved model performance for in-vehicle travel and degraded performance for outdoor walking predictions in the UCI data. The inferior model performance for outdoor walking in the UCI data may be because over 85% of the limited outdoor walking data (a total of approximately 8 hours) came from one of the staff during his one-day tour to the Sea World theme park in San Diego. It is difficult to distinguish walking from the other activities since the tour was associated with brisk walking, frequent short stops, slow walking speed, and potentially high uncertainties in the coding of this type of activity. As expected, most of his walking points were misclassified by the rule-based model as outdoor static (Table 1). Even though most of the thresholds for classification rules were developed based on the HCTLS data, we observed better results from the rule-based model for the other type of time-activity patterns (i.e. indoor, outdoor static, and in-vehicle travel) for the supplemental UCI data, indicating the importance of accurate diary data (in this case more accurate indoor and in-vehicle travel coding in the UCI data) for model development, training, and validation. We did not test whether the performance of the rule-based model would be improved if we refined classification rules using statistics from the UCI data because these data were limited to only three subjects who were indoors 87% of the time and had non-representative outdoor walking data. In addition, model performance may also be influenced by the different GPS devices used in the two dataset; however, we cannot determine this impact based on available data. We suggest avoiding the use of different GPS devices where a single time activity model is to be used for all of the GPS data.

We found that misclassifying between indoor and outdoor static points was one of the most severe problems in both the rule-based and the random forest models. In Southern California, the majority of residential homes are wood structures that do not block satellite signals appreciably. In addition, for outdoor locations adjacent to buildings structures, GPS signals may be reflected by the buildings and result in positional error [24]. Because of the above reasons, the quality of the GPS data did not differ much between indoor and outdoor microenvironments, which makes it difficult to differentiate the two under static conditions. Although we included the scatter patterns of the static clusters to differentiate indoor vs. outdoor static conditions (Figure 5), these measures did not do well as shown in our results. Outdoor walking and in-vehicle travel were also frequently misclassified as outdoor static under low speed conditions (e.g., start or end of the trip near a static cluster). For in-vehicle travel, approximately 80% of the points that were wrongly classified as outdoor static points occurred when the participants stayed in the car without moving (e.g., waiting to pick up someone) or the car moved very slowly approaching or leaving a parking lot (results not shown). The models usually classified vehicle idling longer than two minutes or vehicle moving at very low speed as outdoor static, whereas participants usually reported such conditions as in-vehicle travel. One the other hand, we found that approximately 75% of the outdoor static points that were wrongly classified as in-vehicle travels lasted less than two minutes, indicating that this type of misclassification came from either coding errors (e.g., participants and research staff may have difficulty correctly reporting/coding the start or end of an in-vehicle travel period compared to an outdoor static period) or modeling errors (e.g., the models were incapable of reliably classifying time activity categories near the start or end of an activity).

Three major limitations exist in this study. First, the manually-classified time activity classifications of the HCTLS and UCI data were not error free despite the extensive measures we have taken to minimize errors and enhance the accuracy of the coding (e.g., GPS tracking was combined with a paper diary and in-person interview). Participants in the HCTLS tended to not record short stops or trips such as walking to an adjacent school to pick up a child from school or walking to a nearby corner store [37]. Although follow-up interviews were used to clarify these discrepancies and to finalize the classifications of these missing periods, these data may not reliably capture the precise time of transitions between indoor and outdoor spaces particularly when participants lingered near a building entrance during the transition. Furthermore, even the most compliant participants (including our research staff) may have difficulty correctly documenting the start and end time of an activity because it takes time and effort to record such information on a time activity log and this could interfere with ongoing activities.

The second limitation is that we collected data for 47 participants living in southern Los Angeles County and three staff volunteers living in Irvine, Orange County. Although the participants traveled throughout the region, , we did not target data collection for residents of areas with a more challenging built environment for GPS data collection, such as downtown Los Angeles where there are many tall and high-density buildings which our previous research indicates could result in more GPS signal loss and positional error [24]. Complete loss of satellite signals indoors, however, may not be a drawback because it can help determine whether people are indoors or outdoors. Future work is needed to refine the models so that they can classify time activity patterns in a wide range of built environments.

The third limitation of the study is that our GPS-based time-activity patterns were not comprehensive enough for us to explore more refined time-activity patterns. For example, we had little GPS data for other modes of travel (e.g., bus, biking, and train). Future studies should develop more refined classifications based on a combination of over-sampling of these travel modes and using related supplemental GIS data (e.g., trails and bus and train routes). In addition, we did not look at the impact of different GPS recording intervals on the model performance since the data were very limited for the other time intervals. Only one subject had 1-second interval data (sometimes two seconds due to recording fluctuation); the fluctuation in GPS recordings at other intervals accounted for only about 3.9% of the data. We believe the rule-based model can be easily adjusted to accommodate different recording intervals.

Future work should consider if the performance of the models can be further improved by including supplemental measurement data, GPS diagnostic parameters, and detailed points-of-interest (POI) information. Adding data from a physical activity sensor (pedometer or actigraph) may provide improved discrimination across the indoor and outdoor environments. Certain GPS devices output diagnostic information such as the number of satellites used in the determination of locations, the number of satellites in view, and the dilution of precision which can influence satellite signal quality and positional accuracy. Our previous work has shown that horizontal dilution of precision was a good measure of spatial accuracy of the GPS data and may be helpful in distinguishing indoor vs. outdoor points [24]. Other studies have shown the number of satellites used in the location determination and the number of satellites in view can be used to improve classifications of GPS data [51]. However, our GPS data did not contain such information. Commercial POI data provide information on building and facility type (e.g. school, shopping, restaurant, hospital, and park) and are available from a number of companies (e.g., Garmin) with the main application being in GPS-based tracking and mapping software. Such POI data can help refine models to better classify different microenvironments (e.g. residence, school, restaurant) and time activity patterns (e.g. travel mode) and improve exposure assessment of air pollutants [27].

As we discussed above, this study was limited by input data quality (e.g. error in manually-classified time activity classifications) and quantity (e.g. lack of GPS diagnostic and physical activity data). Despite the limitations, our models showed promising results in identifying time-location patterns in air pollution health studies where exposure levels vary remarkably by location (e.g. indoor, outdoor, and in-transit). With technology advances, the data quality and quantity will be improved significantly in the future research. For instance, some GPS devices (e.g. VGPS-900 from Visiontac™) can voice tag POI, which can improve the manually-classified time activity classifications. In addition, certain physical activity monitors (e.g. GT3X+ activity monitor from ActiGraph™) records both activity level and ambient light - an intriguing parameter for distinguishing indoor vs. outdoor environment. In addition to the ease of use and the minimal learning curve, the random forest model is capable of handling a large number (hundreds to thousands) of variables, which makes it a suitable modeling tool in future studies that may collect many types of measurement data in high temporal resolutions (e.g. GPS, actigraph, and electrocardiogram data). With appropriate data, the model can be easily adapted to classify both time-location and physical activity levels, which will advance the understanding of potential interactions between air pollution exposure and physical activity levels on health outcomes. In addition to air pollution epidemiology, the model can also be applied in other public health fields, such as physical activity and obesity research. More effort is needed to modify and validate the rule-based model before it can incorporate or classify other types of data. But the rule-based model can be a good choice if the researchers have a good understanding of the data and if the decision tree approach cannot capture certain patterns in the data.

## Conclusions

We successfully developed and evaluated two models to identify indoor and in-vehicle travel periods from raw GPS data under free living conditions. The rule-based model classified indoor and in-vehicle travel points reasonably well, but the performance was moderate for outdoor static and outdoor walking predictions. No striking differences were observed between the rule-based and the random forest models. The random forest model was fast and easy to execute, but was likely less robust than the rule-based model when the quality of the training data was poor.

## Abbreviations

DOQQ: Digital orthophoto quarter quadrangle; GPS: Global positioning system.

## Competing interests

The authors declare that they have no competing interests.

## Authors' Contributions

JW is the PI of this study. JW conceptualized the study, participated in study design, method development and data analysis, and drafted the manuscript. CJ conducted the modeling work and data analysis, and helped drafting the manuscript. DH provided the HCTLS GPS time activity dataset, reviewed the manuscript, and revised it critically for important intellectual content. DB and RD helped conceptualize the study and provided important intellectual content for the manuscript. All authors assisted in the interpretation of results and contributed towards the final version of the manuscript. All authors read and approved the final manuscript.

## Supplementary Material

Additional file 1 **Supplementary Material, Tables S1 and S2**. Statistics of the duration of static clusters and periods of movement in each time activity category and results of sensitivity tests of maximum depth in the 10-tree random forest models. Click here for file
