# Supplementary material for: Automated time activity classification based on global positioning system (GPS) tracking data
Source: Environ Health. 2011 Nov 14;10:101. doi: 10.1186/1476-069X-10-101 (PMC3256108; doi:10.1186/1476-069X-10-101)
Supplement: Additional file 1 — Supplementary Material, Tables S1 and S2. Statistics of the duration of static clusters and periods of movement in each time activity category and results of sensitivity tests of maximum depth in the 10-tree random forest models. [file 1476-069X-10-101-S1.DOC]

Table S1. Statistics of the number of points and duration of static clusters and periods of movement in each time activity category of the HCTLS data

|  | Minimum  N (minutes) | 5^th^ quartile  N (minutes) | 25^th^ quartile  N (minutes) | 50^th^ quartile  N (minutes) | 75^th^ quartile  N (minutes) | 95^th^ quartile  N (minutes) | Maximum  N (minutes) |
| --- | --- | --- | --- | --- | --- | --- | --- |
| Indoor | 1 (0.3) | 6 (1.5) | 32 (9.3) | 133 (41.3) | 464 (138.5) | 1403 (812.8) | 8281 (3174.0) |
| Outdoor static | 1(0.3) | 3(0.5) | 6(1.3) | 10(2.3) | 20(4.8) | 92(22.8) | 738(184.3) |
| Outdoor walking | 1(0.3) | 3.0 (0.5) | 10 (2.3) | 21 (5.0) | 37 (9.2) | 89 (22.0) | 251 (63.3) |
| In-vehicle travel | 1(0.3) | 5.0 (1.0) | 18 (4.3) | 37 (9.0) | 70 (17.5) | 166 (42.8) | 1070 (271.8) |

Table S2. Sensitivity tests of maximum depth in the 10-tree random forest models

| Depth |  | HCTLS model^a^ 10-fold cross validation^b^ | | | HCTLS model^a^ evaluated against the full UCI dataset | | | UCI model^c^ 10-fold cross validation^b^ | | | UCI model^c^ evaluated against the full HCTLS dataset | | |
| --- | --- | --- | --- | --- | --- | --- | --- | --- | --- | --- | --- | --- | --- |
|  |  | Sensitivity^d^ | Specificitye^e^ | Precision^f^ | Sensitivity^d^ | Specificitye^e^ | Precision^f^ | Sensitivity^d^ | Specificitye^e^ | Precision^f^ | Sensitivity^d^ | Specificitye^e^ | Precision^f^ |
| 2 | Indoor | 80.4% | 74.3% | 58.2% | 97.9% | 64.8% | 96.4% | 78.8% | 94.6% | 86.7% | 36.2% | 95.8% | 97.7% |
|  | Outdoor static | 25.7% | 87.4% | 41.2% | 11.6% | 97.9% | 16.9% | 63.3% | 90.7% | 70.5% | 33.2% | 74.8% | 7.9% |
|  | Outdoor walking | 69.9% | 93.1% | 61.5% | 34.1% | 99.4% | 45.8% | 63.9% | 94.4% | 59.9% | 91.9% | 65.0% | 8.1% |
|  | In-vehicle travel | 75.4% | 95.2% | 87.0% | 88.8% | 99.3% | 85.5% | 94.2% | 90.5% | 82.1% | 73.9% | 98.7% | 81.7% |
| 3 | Indoor | 73.2% | 81.8% | 64.1% | 75.6% | 91.6% | 98.9% | 79.6% | 99.0% | 97.3% | 45.2% | 92.8% | 96.9% |
|  | Outdoor static | 39.1% | 81.7% | 42.3% | 74.1% | 76.2% | 10.1% | 77.5% | 90.2% | 73.6% | 26.4% | 84.5% | 10.0% |
|  | Outdoor walking | 74.8% | 92.9% | 62.5% | 29.3% | 99.8% | 67.7% | 69.4% | 94.4% | 62.1% | 89.2% | 63.0% | 7.5% |
|  | In-vehicle travel | 74.3% | 96.2% | 89.4% | 88.8% | 99.3% | 85.5% | 93.1% | 92.6% | 85.3% | 72.6% | 98.9% | 83.8% |
| 4 | Indoor | 71.6% | 83.9% | 66.3% | 75.6% | 91.5% | 98.9% | 85.6% | 99.1% | 97.6% | 46.0% | 92.2% | 96.7% |
|  | Outdoor static | 44.0% | 80.7% | 44.0% | 74.3% | 76.4% | 10.3% | 81.2% | 92.6% | 79.3% | 36.8% | 79.1% | 10.4% |
|  | Outdoor walking | 74.0% | 93.9% | 65.5% | 30.9% | 99.6% | 55.0% | 77.2% | 94.6% | 65.3% | 76.6% | 69.9% | 7.9% |
|  | In-vehicle travel | 75.8% | 95.6% | 88.1% | 88.8% | 99.3% | 85.5% | 92.4% | 95.0% | 89.4% | 73.7% | 98.3% | 76.9% |
| 5 | Indoor | 71.3% | 85.0% | 67.9% | 73.5% | 91.0% | 98.8% | 88.2% | 99.2% | 98.1% | 57.0% | 85.4% | 95.1% |
|  | Outdoor static | 45.6% | 81.5% | 45.9% | 47.0% | 83.7% | 9.5% | 82.0% | 94.1% | 83.1% | 33.9% | 82.0% | 10.9% |
|  | Outdoor walking | 74.9% | 94.5% | 68.3% | 24.8% | 99.9% | 78.3% | 79.9% | 94.8% | 67.1% | 72.4% | 77.3% | 9.7% |
|  | In-vehicle travel | 79.3% | 94.9% | 87.0% | 89.8% | 88.7% | 26.0% | 93.4% | 95.1% | 89.9% | 72.8% | 98.5% | 78.6% |
| 6 | Indoor | 73.8% | 84.5% | 67.9% | 71.5% | 89.2% | 98.5% | 90.9% | 99.5% | 98.7% | 51.6% | 86.0% | 94.9% |
|  | Outdoor static | 45.9% | 83.3% | 48.6% | 37.4% | 82.8% | 7.3% | 86.4% | 94.9% | 85.6% | 31.9% | 83.6% | 11.3% |
|  | Outdoor walking | 75.0% | 95.3% | 71.7% | 29.3% | 99.7% | 63.9% | 79.5% | 96.1% | 72.8% | 72.2% | 70.2% | 7.6% |
|  | In-vehicle travel | 81.1% | 94.6% | 86.5% | 91.2% | 87.8% | 24.9% | 93.7% | 95.5% | 90.5% | 66.7% | 98.7% | 79.8% |
| 8 | Indoor | 78.4% | 85.8% | 71.1% | 78.5% | 84.2% | 98.0% | 93.9% | 99.8% | 99.4% | 66.1% | 76.0% | 93.2% |
|  | Outdoor static | 51.5% | 87.2% | 58.1% | 23.6% | 90.1% | 8.0% | 89.9% | 96.6% | 90.3% | 29.5% | 84.4% | 11.0% |
|  | Outdoor walking | 78.3% | 95.9% | 74.9% | 26.5% | 99.9% | 78.6% | 81.2% | 97.6% | 81.5% | 57.8% | 82.3% | 9.9% |
|  | In-vehicle travel | 84.6% | 94.7% | 87.2% | 88.3% | 86.8% | 22.8% | 95.5% | 95.3% | 90.3% | 60.2% | 98.8% | 79.7% |
| 10 | Indoor | 81.7% | 89.0% | 76.8% | 72.8% | 86.0% | 98.1% | 95.8% | 99.7% | 99.4% | 68.6% | 72.3% | 92.6% |
|  | Outdoor static | 61.8% | 90.2% | 68.5% | 46.4% | 77.7% | 7.0% | 92.7% | 97.9% | 93.9% | 23.7% | 87.1% | 10.7% |
|  | Indoor | 84.1% | 96.5% | 79.3% | 26.8% | 99.9% | 81.2% | 85.2% | 98.2% | 86.3% | 58.8% | 81.5% | 9.7% |
|  | Outdoor static | 88.5% | 95.5% | 89.4% | 87.8% | 94.4% | 41.1% | 96.3% | 96.0% | 91.7% | 54.3% | 98.9% | 79.9% |
| 15 | Indoor | 90.9% | 95.8% | 90.6% | 75.3% | 79.8% | 97.3% | 97.2% | 99.5% | 98.9% | 72.4% | 67.8% | 91.8% |
|  | Outdoor static | 84.6% | 95.9% | 87.6% | 37.7% | 77.9% | 5.8% | 95.9% | 98.4% | 95.6% | 19.9% | 89.4% | 10.9% |
|  | Indoor | 93.2% | 98.3% | 89.4% | 18.9% | 99.8% | 63.6% | 88.0% | 98.6% | 89.2% | 56.4% | 82.9% | 10.0% |
|  | Outdoor static | 94.9% | 97.5% | 94.2% | 83.9% | 96.7% | 53.0% | 96.3% | 97.5% | 94.6% | 52.4% | 99.0% | 80.6% |
| No restriction | Indoor | 94.6% | 98.0% | 95.4% | 81.4% | 74.1% | 96.8% | 97.5% | 99.5% | 98.8% | 73.3% | 66.1% | 91.6% |
|  | Outdoor static | 93.0% | 97.3% | 92.1% | 32.9% | 84.1% | 7.0% | 96.4% | 98.4% | 95.5% | 18.8% | 90.1% | 11.1% |
|  | Outdoor walking | 94.7% | 99.0% | 93.7% | 18.1% | 99.9% | 69.7% | 88.7% | 98.7% | 90.1% | 53.1% | 82.9% | 9.5% |
|  | In-vehicle travel | 95.9% | 98.4% | 96.3% | 84.1% | 96.5% | 51.8% | 96.1% | 97.7% | 95.1% | 51.5% | 99.1% | 82.3% |

^a^The model was developed based on all outdoor static, outdoor walking, in-vehicle travel and randomly selected 30,000 indoor points ^b^The reported validation results were the averages from repeated 10-fold cross validation.

^c^The model was developed based on all outdoor static, outdoor walking, in-vehicle travel and randomly selected 5,000 indoor points from the supplemental UCI data.

^d^Sensitivity was calculated as true positive estimation/(true positive estimation + false negative estimation).

^e^Specificity was calculated as true negative estimation/(true negative estimation + false positive estimation).

^f^Precision was calculated as true positive estimation/(true positive estimation + false positive estimation).
